# Risk factors associated with *Trypanosoma cruzi* exposure in domestic dogs from a rural community in Panama

**DOI:** 10.1590/0074-02760150284

**Published:** 2015-11

**Authors:** Azael Saldaña, José E Calzada, Vanessa Pineda, Milixa Perea, Chystrie Rigg, Kadir González, Ana Maria Santamaria, Nicole L Gottdenker, Luis F Chaves

**Affiliations:** 1 Instituto Conmemorativo Gorgas de Estudios de Salud, Departamento de Parasitología, Ciudad de Panamá, República de Panamá; 2 Universidad de Panamá, Facultad de Medicina, Centro de Investigación y Diagnóstico de Enfermedades Parasitarias, Ciudad de Panamá, República de Panamá; 3 Universidad de Panamá, Facultad de Medicina Veterinaria, Ciudad de Panamá, República de Panamá; 4 University of Georgia, College of Veterinary Medicine, Department of Veterinary Pathology, Athens, GA, USA; 5 Nagasaki University, Institute of Tropical Medicine, Nagasaki, Japan; 6 Universidad Nacional, Escuela de Medicina Veterinaria, Programa de Investigación en Enfermedades Tropicales, Heredia, Costa Rica

**Keywords:** *Trypanosoma cruzi*, *Canis familiaris*, *Rhodnius pallescens*, *Attalea butyracea*, Chagas disease, Panama

## Abstract

Chagas disease, caused by *Trypanosoma cruzi* infection, is a zoonosis of humans, wild and domestic mammals, including dogs. In Panama, the main *T. cruzi* vector is * hodnius pallescens*, a triatomine bug whose main natural habitat is the royal palm, *Attalea butyracea*. In this paper, we present results from three *T. cruzi* serological tests (immunochromatographic dipstick, indirect immunofluorescence and ELISA) performed in 51 dogs from 24 houses in Trinidad de Las Minas, western Panama. We found that nine dogs were seropositive (17.6% prevalence). Dogs were 1.6 times more likely to become *T. cruzi* seropositive with each year of age and 11.6 times if royal palms where present in the peridomiciliary area of the dog's household or its two nearest neighbours. Mouse-baited-adhesive traps were employed to evaluate 12 peridomestic royal palms. All palms were found infested with *R. pallescens* with an average of 25.50 triatomines captured per palm. Of 35 adult bugs analysed, 88.6% showed protozoa flagellates in their intestinal contents. In addition, dogs were five times more likely to be infected by the presence of an additional domestic animal species in the dog's peridomiciliary environment. Our results suggest that interventions focused on royal palms might reduce the exposure to *T. cruzi* infection.

Chagas disease, a parasitic infection caused by *Try-panosoma cruzi, *has a predominantly enzootic cycle in Panama (Whitlaw & Chaniotis 1978). In most cases, human infection occurs when sylvatic infected vectors are attracted to lights, food sources or other factors related to human dwellings (Romaña et al. 2003). This transmission scenario differs from that reported in some Central and South American countries, where vectors colonise human dwellings and where domestic mammals, including dogs, are important *T. cruzi* reservoirs (Moncayo & Silveira 2009, Esch & Petersen 2013, Castillo-Neyra et al. 2015). Numerous studies have found domestic dogs infected with *T. cruzi* across endemic areas ranging from southern United States of America to Argentina (Gürtler et al. 2007). The reported prevalence varies widely (1.42-92%), depending on ecoepidemiological and sociocultural factors (Crisante et al. 2006, Estrada-Franco et al. 2006, Gürtler et al. 2007, Kjos et al. 2008, Romero-Peñuela & Sánchez-Valencia 2008, Bonfante-Cabarcas et al. 2011, Manrique-Abril et al. 2012, Lizundia et al. 2014). Generally, canine infections are more prevalent than human infections, a fact likely related to oral *T. cruzi* transmission, a more efficient route of infection and apparently common to many mammals (Barretto et al. 1978, Roellig & Yabsley 2010), and also the fact that dogs often sleep near houses and may come in greater contact with peridomiciliary vectors. Dogs are also considered natural sentinels (Castañera et al. 1998, Castillo-Neyra et al. 2015) and a biological barrier for human transmission (Pineda et al. 2011). In Panama, *T. cruzi *dog infection has also been reported in some active transmission areas (Romaña et al. 2003, Pineda et al. 2011, Fung et al. 2014). However, the prevalence of*T. cruzi-*infected dogs has not been studied in association with spatially explicit risk factors that could help to improve current, or to propose new control, measures. The objective of this study was to evaluate the exposure of dogs to*T. cruzi* infections in the rural community of Trinidad de Las Minas, Republic of Panama in relation to local and spatially explicit risk factors.

## MATERIALS AND METHODS

*Study site* - We studied *T. cruzi *exposure in dogs from 24 households in Trinidad de Las Minas (8º46'32''N 79º59'45''W), district of Capira, western Panama, a place where we have previously studied the epidemiology and control of cutaneous leishmaniasis (Saldaña et al. 2013). The 24 households were a clustered subset from 128 households in Trinidad de las Minas with similar ecoepidemiological conditions that were evaluated as part of a sandfly control trial using insecticide thermal fogging and where residents provided informed consent to monitor changes in sandfly abundance (Chaves et al. 2013), but also for the collection of blood samples from domestic animals (Calzada et al. 2015, González et al. 2015). In Trinidad de las Minas around 90% of the houses have dogs and 22 out of the 24 houses we studied had dogs. The district of Capira is generally considered endemic for Chagas disease; however no human seroprevalence studies have been conducted in the area of Trinidad de Las Minas. Climate in this community is unimodal, with a rainy (April-November) and dry (December-March) season. Monthly rain ranges from 28-570 mm^3^. Temperature is nearly constant with a year-round 26ºC average. Characteristics of the study site were described in detail by Calzada et al. (2013).

*Dog sampling* - Inhabitants from 24 selected houses at Trinidad de Las Minas were asked the age and sex of each dog they owned. Dog age was confirmed by dentition and tartar deposition patterns (Calzada et al. 2015). A physical examination was performed and dogs were combed and visually inspected to evaluate ectoparasite infestation by fleas, lice or ticks. General body condition of each dog was assessed visually and by touch (spine and ribs) based on the scale developed by Baldwin et al. (2010). This information was also evaluated as a potential risk factor for exposure to *T. cruzi *when analysed in light of the results from the serological tests. Dogs were manipulated in the presence of their owners. Blood samples (3 mL) were obtained by puncture of the cephalic vein and collected into sterile serum tubes without anticoagulant. Two millilitres of blood were centrifuged. The serum fraction was collected for serological analysis and the remaining blood was resuspended with liver infusion tryptose medium and incubated at 27ºC, using the protocol presented by Vásquez et al. (1997). Cultures were checked weekly during two months for parasites presence. Serum from each dog was obtained by centrifugation at 2,000 rpm for 20 min and stored at -20ºC until use. Serum samples were analysed for anti-*T. cruzi* antibodies by a rapid test (*Trypanosoma cruzi*Detect™, InBios International Incorporated, USA), a partially modified commercial recombinant ELISA (ELISA Chagastest, Wiener lab, Argentina) using an anti-dog IgG peroxidase conjugated diluted 1:2000 (Sigma No A6792) and an indirect immunofluorescence antibody test (IFAT) with a local* T. cruzi*isolate (Burunga strain) as antigen. The first two tests are based on the use of recombinant antigens and, according to the manufacturers, these tests do not cross-react with other trypanosomatids, including *Leishmania* spp and *Trypanosomarangeli*. The rapid test was carried out according to the manufacturer's instructions. It has a specificity of at least 94% and a sensitivity of at least 96% (Cardinal et al. 2006). The test was only considered positive when a second defined line besides the control line appeared on the test field. The intensity of the colour line was not interpreted in this study. For the ELISA, we replaced the anti-human IgG conjugate with an anti-dog IgG conjugate. We previously validated this ELISA kit with a panel of 10 positive and 10 negative canine sera from Chagas disease endemic areas in Panama. This test was optimised as follows: serum dilution (1:25), incubation time (30 min), dilution of anti-dog IgG conjugate (1:2000 Sigma No A6792) and signal development. For the IFAT, our controls also were positive and negative dog sera from Chagas disease endemic areas in Panama. The cut-off titre was 1:40, which was determined with a panel of 10 positive and 10 negative canine sera.

*Ecological risk factors for exposure to T. cruzi* - We collected information on potential ecological risk factors associated with canine Chagas disease. For each household we estimated: a housing destituteness index, which quantified how different elements of housing construction could render a house a suitable habitat for triatomine bugs (and insects in general), a peridomicile index, that quantified the presence of elements that could serve as refuge for triatomine bugs, for example palms and a vegetation index, that measured natural vegetation vertical structure, i.e., whether a site had many trees or was a pasture. We also quantified species richness, i.e., the number of species of domestic and wildlife mammals. In addition, we developed an index of domestic animal abundance which, based on a principal components analysis, weighted the abundance of different domestic species belonging to a household. Similarly, we developed an index for wild animal presence that weighted the commonness of different wildlife mammal species sighted by householders. A detailed description of data collection and the estimation of each index was presented by Chaves et al. (2013).

Since the dominant triatomine vector species in our study area is *Rhodnius pallescens*, a sylvatic species whose main natural habitat is the royal palm, *Attalea butyracea *(Pipkin 1968, Whitlaw & Chaniotis 1978, Gottdenker et al. 2011), we also considered the presence of this palm species within the peridomicile (here defined as the area within a 50 m radius circumference around each household) of a focal household or in the peridomicile of any of the two households closest to the focal household. The 50 m radius was chosen to account for both sandfly and triatomine bug dispersal. For sandflies, dispersal distances rarely are over 50 m (Chaves et al. 2013) and field measurements recorded for *Rhodnius prolixus *(Gómez-Núñez 1969) suggest *Rhodnius* spp movement in peridomiciles unlikely exceeds 15 m. Similarly, data for *R. pallescens* from flight mills has found median flight distances for adults of 50 m (Castro et al. 2014). The presence of triatomine bug palm infestation was determined using mouse-baited-adhesive traps, which can detect adults and nymphal instars (Noireau et al. 2002), three traps by palm for one night. The intestinal contents of all captured adults triatomines were analysed microscopically for the presence of flagellated protozoa.

*Sensitivity and specificity for the serological tests indicative of T. cruzi exposure* - Sensitivity, the accurate diagnosis of true infections and specificity, the proper diagnosis of lack of an infection, are generally assessed in the presence of a “gold standard” (Lalkhen & McCluskey 2008), for example, the direct observation of a parasite or its DNA amplification *via* polymerase chain reaction (Altman & Bland 1994). For Chagas disease (i.e., *T. cruzi* infections), due to the low parasitaemia observed in indeterminate and chronic infections, the Pan American Health Organization recommends the confirmation of the diagnosis by at least two serological methods (Carvalho et al. 1993), a criterion that we used as a gold standard to estimate sensitivity and specificity of the serological diagnostic tests following the steps described by Lalkhen and McCluskey (2008). We also estimated Cohen's kappa coefficient (Cohen 1960), a measurement of agreement between diagnostic tests, for all possible combinations of two serologic diagnostic methods and for all the diagnostic methods at once.

*Risk factors for T. cruzi seropositive diagnostic* - We first performed a household level analysis of risk factors associated with *T. cruzi* seropositive dogs, where dogs were assigned a seropositive status when they were positive by two or more diagnostic tests. For this purpose, we employed maximum likelihood Binomial Generalized Linear Models (Bin-GLMs) (Faraway 2006). In a first round of model selection we compared models with the same number of parameters, but with alternative risk factors. Briefly, all models included the housing destituteness index and the vegetation index, but considered the presence of domestic, which were censused by us and included dogs, cats, horses, chickens and parrots and wildlife animals, which were self-reported by householders and included sloths, opossums, porcupines, voles, bats, squirrels, monkeys and birds, by considering either their species richness or their abundance/presence indices. Similarly, we either considered the peridomiciliary index or palm presence in the peridomicile of a focal household or palm presence in either the focal household of the two closest neighbouring houses to a focal household. The best model selected in this first round was then further simplified employing a process of backward elimination (Faraway 2004). We selected *T. cruzi* seropositivity risk factors by using the Akaike Information Criterion (AIC), a criterion that both considers model likelihood and parameter number, because models were not nested during the first round of risk factor selection, i.e., not comparable *via* likelihood ratio tests since models did not shared the same variables (Venables & Ripley 2002). Also, the alternative covariates we defined before are collinear, which can lead to problems in parameter estimation of linear models (Faraway 2004). Given the spatial nature of our data, we also tested the lack of spatial autocorrelation in the best model residuals, employing Moran's I test (Venables & Ripley 2002). After the selection of relevant household risk factors for*T. cruzi* seropositive dogs, we performed an analysis considering information about potential individual based risk factors employing Logistic Generalized Estimating Equations Models (Log-GEEM). We employed Log-GEEM given the nature of the data, dogs belonging to the same household are not independent observations, a fact constraining the use of regression tools that assume observation independence (Chaves 2010). We based our inferences on a sandwich estimator to obtain robust standard errors (SE), since naïve SEs are appropriate only when an appropriate correlation structure is specified (Faraway 2006). We thus assumed independence in the correlation structure of the model, given the robustness of GEEMs to provide reliable estimates using the robust SEs from the sandwich estimator (Venables & Ripley 2002). We first specified a full model that included the best household level risk factors for *T. cruzi* seropositive dogs and the individual level factors that were variable in the dogs we studied. This model was simplified using a procedure similar to the one employed for the Bin-GLMs, but exclusively based on the quasi-likelihood information criterion (Pan 2001) the GEE analog to AIC.

*Ethics* - This study was evaluated and approved by the National Review Board, National Bioethics Committee of Research (CNBI), Gorgas Memorial Institute for Health Studies (ICGES), Panama (561/CNBI/ICGES/06), and from ICGES Institutional Animal Care and Use Committee (2006/02). The study was in accordance with law No. 23 of 15 January 1997 (Animal Welfare Assurance) of Republic of Panama.

## RESULTS

**Fig. 1A:**
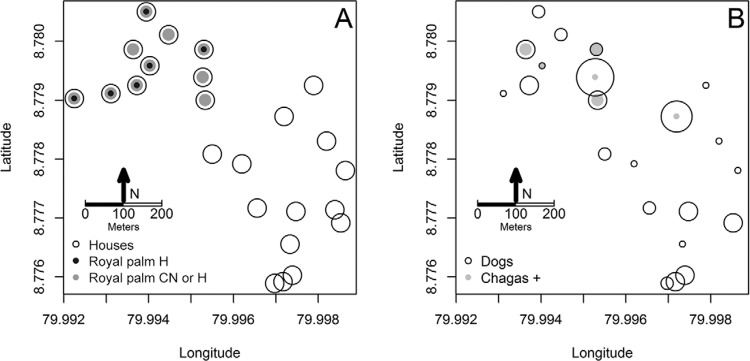
royal palms and *Trypanosoma cruzi *seropositive dogs; B: study houses indicating whether their peridomicile (area within a 50 m radius circumference around the house) or that of the two closest neighbouring houses had the royal palm *Attalea butyracea*. Number of *T. cruzi* seropositive dogs (based on positive diagnosis from at least 2 tests) and all dogs per household in this panel symbol size in the inset legend corresponds to one individual. CN: in the peridomicile of the two closest neighbouring houses; H: in the house peridomicile.

**Fig. 2:**
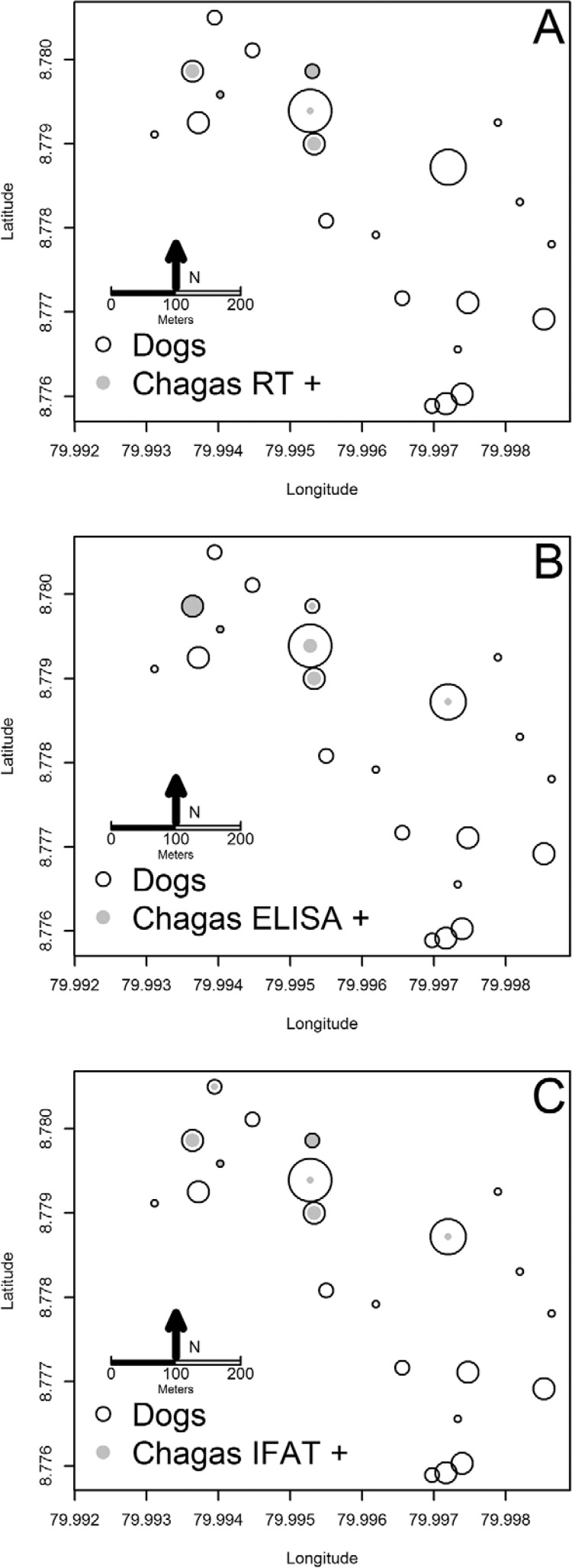
number of *Trypanosoma cruzi* seropositive dogs per household according to different diagnostic tests: rapid test (RT) (A) ELISA Winner (B) and immunofluorescence antibody test (IFAT) (C). Each panel also shows the total number of dogs per household. In each legend symbol size corresponds to two individuals.

The 24 houses we surveyed (Fig. 1A) had 52 dogs, but we collected information and samples from 51, since one of the dogs was less than one month old and still weaning. All dogs were mixed breeds. The average (± SD) age of the dogs was 3.07 ± 2.94 years, 17 were females and 34 males. Most dogs (94%) had ectoparasites (ticks and/or fleas) attached to their skin, 43% had a poor physical condition and 7% slept inside the houses. Regarding *T. cruzi* infection, eight dogs were seropositive according to the rapid test, 10 by the modified ELISA and 10 by homemade IFAT. Nine dogs were considered seropositive by the composite gold standard (seropositivity in 2 or more tests), representing a seroprevalence of 17.6% (Fig. 1B). The spatial location of households where seropositive dogs were diagnosed by each test is depicted in Fig. 2. The sensitivity and specificity of the different serological tests are shown inTable I. The most sensitive and specific test was the Rapid test, followed by the IFAT and the modified ELISA. The inter-agreement between all diagnostic tests (Table II) was substantial according to the scale by Landis and Koch (1977) and between the paired tests, with the exception of the Rapid test - IFAT pair (Table II), whose value above 0.8 can be interpreted as an almost perfect agreement on *T. cruzi *serodiagnosis (Landis & Koch 1977). Thus, in general, it can be affirmed that the diagnostic tests were in agreement on positive and negative diagnostics. None of the blood cultures were positive for hemoflagellates after two months of microscopic observation.

**TABLE I:** Sensitivity and specificity of serological diagnostic tests for*Trypanosoma cruzi *exposure

Diagnostic test	Sensitivity (%)	Specificity (%)
Rapid test	100	98
ELISA Winner	80	95
IFAT	90	98

seropositivity was used by at least two tests as a gold standard. IFAT: immunofluorescence antibody test.

**TABLE II:** Kappa coefficient of agreement between the serological diagnostic tests for *Trypanosoma cruzi *exposure

Diagnostic test	Kappa	Z	p
Rapid test - ELISA Winner	0.731	5.27	< 0.00001^*a*^
Rapid test - IFAT	0.865	6.24	< 0.00001^*a*^
IFAT - ELISA Winner	0.751	5.36	< 0.00001^*a*^
Rapid test - IFAT - ELISA Winner	0.781	9.67	< 0.00001^*a*^

*a*: statistically significant (p < 0.05); IFAT: immunofluorescence antibody test.

Royal palms were clustered around houses in the northern section of our study site (Fig. 1A), as were the greatest number of*T. cruzi* seropositive dogs positive by at least two diagnostic tests (Fig. 1B). Twelve palm trees were sampled for triatomine bugs in the peridomicile areas of houses (and their closest neighbours) where dogs were seropositive. All of the palms had *R. pallescens* (100% infestation index),with a capture rate of 8.5 triatomine/trap/night and an average [± standard deviation (SD)] of 25.50 ± 14.26 specimens captured per palm, specifically with 18.83 ± 15.08 nymphs/palm and 4.67 ± 4.92 adults/palm. Here, it is important to note that the low variability (measured by the SD) to mean ratio in triatomine bugs imply that they were uniformly distributed across the palms independently of their age, but randomly distributed regarding their age, where the ratio was close to one (Morisita 1962). Of 35 adult bugs captured, 31 (88.6%) had protozoan flagellates in their intestinal contents during microscopic examination. The presence of palms in the peridomicile of a focal household or the peridomicile of any of its two closest neighbouring houses was a significant risk factor for a dog being *T. cruzi* seropositive at the household level (Table III). The other significant factor associated with *T. cruzi* seroprevalence patterns across the surveyed houses was the number of domestic animal species owned by a given house (Table III). At the household level, royal palm presence increased the odds of dogs being seropositive to *T. cruzi* by 11 times and each domestic mammal species in a household increased the likelihood of *T. cruzi* seropositivity in dogs belonging to that household by four times (Table IV). The residuals of the best model were spatially independent (Table IV), ensuring a statistically sound inference (Venables & Ripley 2002). The only factors that were variable enough across the dogs we surveyed to be evaluated as risk factors in the population we studied were dog physical condition, age and sex. The simplification of a model considering these three factors, as well as the presence of royal palms in the peridomicile of the household (and/or the 2 closest neighbours) where a dog belonged and domestic animal species richness, showed that physical condition and sex were not important risk factors for *T. cruzi* seroprevalence (Table V). The best logistic generalised estimating equations model (Table VI) found that odds of an individual dog being*T. cruzi* seropositive increased 1.59 times with each year of age, 11.5 times when there are royal palms in the household (or 2 closest neighbouring households) and about five times by the presence of a domestic animal species other than a dog.

**TABLE III:** Model selection of risk factors for *Trypanosoma cruzi* seropositive reactions in dogs at the household level in Trinidad de Las Minas, western Republic of Panama

Selection round	Risk factors	AIC	ΔAIC
0^*a*^	Palms + VI + HP + DAI + WPI	37.10	11.37
	PI + VI + HP + DAI + WPI	35.72	9.99
	PI + VI + HP + DSR + WSR	33.28	7.55
	Palms + VI + DI + DSR + WSR	31.62	5.89
	Npalms + VI + HP + DAI + WPI	30.52	4.79
	Npalms + VI + DI + DSR + WSR	26.81	1.08
1^*b*^	**Npalms + DSR**	**25.73**	--

*a*: models with the same number of parameters, but alternative covariates; *b*: final model from the stepwise backward elimination of selection round 0; AIC: Akaike Information Criterion; DAI: domestic animal abundance index; DSR: domestic animal species richness; HP: housing destituness; Npalms: palms in the peridomicile of the focal household and/or any of its two closest neighbours; Palms: palms in the peridomicile; PI: peridomicile index; VI: vegetation index; WPI: wild animal presence index; WSR: wildlife animal species richness; ΔAIC: is the difference between each model AIC with that of the model with minimum AIC. The final best model (minimum AIC) is bolded.

**TABLE IV:** Odds ratios (OR) and parameter estimates for the best binomial generalised linear model for risk factors associated with*Trypanosoma cruzi s*eropositive reactions in domestic dogs at the household level in Trinidad de Las Minas, western Republic of Panama

Parameter	OR (95% CI)	Estimate	SE	Z	p
Intercept	1	-7.25	2.27	-3.18	0.001^*a*^
Palms in focal household and/or two closest neighbouring houses	11.22 (1.56-236.60)	2.42	1.17	2.06	0.039^*a*^
Domestic animal species richness	3.97 (1.41-16.99)	1.38	0.61	2.26	0.024^*a*^
Moran's I of the residuals^*b*^	-	-0.27	-	-	0.923

*a*: statistically significant (p < 0.005);*b*: the inference for the Moran's I is based on a MonteCarlo simulation; CI: confidence interval; SE: standard error.

**TABLE V:** Model selection for risk factors associated with *Trypanosoma cruzi* seropositive reactions in dogs from Trinidad de Las Minas, western Republic of Panama

Selection round	Risk factors	QIC	ΔQIC
0^*a*^	Npalms + DSR + dog poor physical condition, dog sex, dog age	31.55	1.40
1	Npalms + DSR + age + sex	30.76	0.61
2	**Npalms + DSR + age**	**30.15**	-

*a*: full model; DSR: domestic animal species richness; Npalms: palms in the peridomicile of the focal household and/or any of its two closest neighbours; QIC: Pan's quasi-likelihood information criterion; ΔQIC: difference between each model QIC with that of the model with minimum QIC. Best model is bolded.

**TABLE VI:** Odds ratios (OR) and parameter estimates for the best logistic generalised estimating equations model for risk factors associated with*Trypanosoma cruzi s*eropositive reactions in domestic dogs from Trinidad de Las Minas, western Republic of Panama

Parameter	OR	Estimate	Naïve SE	Naïve Z	Sandwich SE	Sandwich Z
Intercept	-	-9.890	2.680	-3.690	3.281	-3.014
Age	1.586	0.461	0.170	2.715	0.188	2.454
Palms in focal household and/or two closest neighbouring houses	11.594	2.450	1.085	2.259	0.957	2.561
Domestic animal species richness	4.978	1.605	0.620	2.587	0.792	2.027

SE: standard error.

## DISCUSSION

The role of domestic dogs in the epidemiology of Chagas disease has been studied extensively in communities where triatomine bugs colonise peridomestic and domestic environments (Castañera et al. 1998, Gürtler et al. 2007, Castillo-Neyra et al. 2015). Nevertheless, studies on dog infections with *T. cruzi *in communities where vectors sporadically invade houses from nearby biotopes, such as *R. pallescens* from palm trees, are relatively rare.

In this study, canine *T. cruzi *infection was confirmed in nine of 51 (17.6%) evaluated dogs from the rural community of Trinidad Las Minas. This village is located in a mountainous region surrounded by abundant forest remnants, where*T. cruzi* animal reservoirs (González et al. 2015) and royal palms are common. This rural settlement has been recently identified as highly endemic for cutaneous leishmaniasis (Saldaña et al. 2013), a vector-borne parasitic disease also associated with tropical humid forests and with high seroprevalence (47%) of *Leishmania panamensis *infection in dogs (Calzada et al. 2015).

Previous studies conducted in other communities located in western province of Panama have reported *T. cruzi* prevalence in domestic dogs as 37% (Romaña et al. 2003), 11.1% (Pineda et al. 2011) and 8.97% (Fung et al. 2014). However, in these studies the association of infected dogs with spatially explicit risk factors for the transmission of *T. cruzi *infection was not assessed. Here we show that the odds of a dog being seropositive for *T. cruzi *increased 1.6 times with each year of age (Table IV). This tendency of increased age associated with *T. cruzi*seroprevalence has been previously reported in many settings in Latin America and could reflect cumulative exposure to parasitic infections (Estrada-Franco et al. 2006, Gürtler et al. 2007, Pineda et al. 2011), similar to the patterns observed for cutaneous leishmaniasis parasites at the study area (Calzada et al. 2015). Most dogs in the studied community of Trinidad de Las Minas are watchdogs that stay most of the time outside and/or around houses. In this peridomestic environment, dogs probably were exposed to *T. cruzi* by eating or chewing infected triatomine bugs from nearby royal palms. Dog infection due to ingestion or contact with infected wild reservoirs, such as opossums, is another possibility, although less likely. Thus, it is reasonable to believe that older dogs have had more exposure with these sources of infection.

No sex-related or body condition differences in the *T. cruzi*seropositive dogs was observed (Table V). However, it is important to consider that 43% of the evaluated dogs were in poor physical condition, a status that could lead to higher rates of *T. cruzi*infectivity to uninfected triatomine bugs that reach the houses (Petersen et al. 2001). Nevertheless, a high percentage (88.6%) of adult triatomines collected on the evaluated royal palms was already infected with trypanosomatids. A similar result was confirmed by molecular techniques in another endemic neighbouring district, where 72.7% of *R. pallescens* entering houses were infected with *T. cruzi*(Calzada et al. 2006). Additionally, we did not find dogs able to generate positive *T. cruzi* blood cultures, suggesting that parasitaemia of the evaluated seropositive dogs was low, most probably because seropositive dogs were chronically infected. The low sensitivity of blood cultures and molecular methods for detecting*T. cruzi* infections during the human chronic phase has been previously reported in Panama (Sousa 1972,Calzada et al. 2006, Garisto-Risco et al. 2009). During physical exams, no evident symptoms of acute Chagas disease were observed in the evaluated dogs. Although acute Chagas disease cases in dogs are rarely reported in Panama, we have observed sporadic fatal cases from suburban areas in western Panama (Samudio et al. 2007). Trinidad de Las Minas is a rural and poor community where no veterinary services are available and no regular surveillance or preventive measures for dog diseases are undertaken by local authorities. Generally, the microclimatic conditions of temperature and humidity needed for *R. pallescens *survival for several weeks are not present in human dwellings. This observation is confirmed by the inability of this species to successfully colonise houses (Calzada et al. 2006, Hurtado et al. 2014). These considerations suggest that domestic dogs play a limited role in the spread of*T. cruzi* in endemic areas of Panama where *R. pallescens* is the main vector. However, dogs can be suitable sentinels for *T. cruzi* epidemiological surveillance in Chagas disease endemic areas (Castañera et al. 1998, Castillo-Neyra et al. 2015), given the easiness to measure infection exposure through serological tests.

In addition, dogs living in households near royal palms had an 11-fold increased odds of *T. cruzi* seropositivity (Table IV). Infestation of palm trees by triatomines has been considered a risk factor for the transmission of Chagas disease in Panama and other regions of South America (Whitlaw & Chaniotis 1978, Romaña et al. 2003, Lima et al. 2008, Angulo et al. 2012, Ricardo-Silva et al. 2012,Hurtado et al. 2014). Royal palms are abundant in the community of Trinidad de Las Minas, yet in the subset of houses we studied, they were clustered towards the north. The entomological evaluation conducted during this study showed that all the palms near the evaluated houses were infested with *R. pallescens*. Also, results from our palm sampling showed that palms can contain many triatomines. In this sense, it is important to mention that during the development of this study, three *R. pallescens* adults were found in houses by homeowners. As suggested in a related study from this region of Panama (Hurtado et al. 2014), Chagas disease control requires the implementation of specific interventions for reducing or eliminating *R. pallescens *populations established on royal palms from peridomestic areas.

Finally, we found that dogs were about five times more likely to be *T. cruzi*seropositive with each domestic animal species present in the household peridomicile in addition to dogs, suggesting that the presence of increased numbers of domestic animal species increase the infection risk, unlike predictions of a “dilution effect” where transmission is expected to decrease with species richness (Keesing et al. 2006). This scenario can be partially explained by an increase in the arrival/activity of triatomines attracted by additional sources for bloodmeals in the peridomestic environment (Rabinovich et al. 2011). In addition, the abundance and richness of domestic animals was positively associated. Thus, the presence of less frequent domestic animals, i.e., horses, cats and parrots, was associated with a larger abundance of dogs and chickens, which might indicate domestic animal abundance, played a role in shaping *T. cruzi* risk exposure patterns. In a recent study, the epidemiological role of domestic animals, especially chickens, was associated with an increased risk of transmission for Chagas infection in competent hosts, even though chickens themselves are not competent hosts for *T. cruzi* (Gürtler et al. 2014). Similar patterns where the “dilution effect” was not observed at the local scale of transmission have been reported for West Nile virus (Loss et al. 2009) and further highlight the role that blood foraging and host accessibility might have on the potential association between host biodiversity and pathogen transmission (Chaves et al. 2007, 2010, Rabinovich et al. 2011).

We must stress that our results are focused on *T. cruzi*-infected dogs and that it is still necessary to determine if similar risk factors are important for humans. In this sense, the presence of *T. cruzi* in dogs at Trinidad de Las Minas raises awareness regarding the potential for human transmission of this parasite in this village that also has high cutaneous leishmaniasis infection prevalence (Saldaña et al. 2013). It is therefore important that health authorities carry out necessary activities to determine the prevalence, management and prevention of Chagas infection among their inhabitants as well as in other nearby communities with similar ecoepidemiological features.
